# Effects of self-regulation strategies on EFL learners’ language learning motivation, willingness to communication, self-efficacy, and creativity

**DOI:** 10.1186/s40359-024-01567-2

**Published:** 2024-02-15

**Authors:** Tingting Zhang

**Affiliations:** Teaching and Research Section of English, Zhujiang College, South China Agricultural University, 510000 Guang Zhou, Guang Dong, China

**Keywords:** Creativity, Language learning motivation, Self-efficacy, Self-regulation strategies, Willingness to communication

## Abstract

This study tried to know how self-regulation strategies (SRS) affected the motivation, self-efficacy, willingness to communicate (WTC), and creativity of Chinese EFL learners as they learned English language. To do this, eighty intermediate EFL students were selected and divided into two groups: experimental and control. Four questionnaires were then used to assess the participants’ motivation for language acquisition, WTC, self-efficacy, and creativity prior to the intervention. Subsequently, SRS was used to treat the experimental group (EG), whereas a traditional instruction was given to the control group (CG) without the use of SRSs. Four questionnaires were given again as post-tests following a 15-session treatment to gauge how the treatment affected the students’ motivation for language acquisition, WTC, self-efficacy, and creativity. Based on the study’s results, the EG considerably outstripped the CG on each of the four post-tests. The findings of this research have some implications for researchers, English teachers, EFL learners, and other stakeholders.

**Supplementary Information:**

The online version contains supplementary material available at 10.1186/s40359-024-01567-2.

## Introduction

Language learners face scholastic difficulties during their schooling, including low grades, stress related to their studies, and decreased motivation [1]. The widespread consensus is that in order to address these issues, proper solutions should be applied and modified. Language learners may have mental health issues if these issues are not addressed. Researchers in EFL educational situations have given this topic particular attention because of how important it is to support mental health in learning environments [2]. Self-regulatory learning is one of the ideas in modern cognitive and academic psychology.

The methods and outcomes of teaching and learning a language depend heavily on self-regulation [3]. Self-regulated learning (SRL) has several definitions. Language learners employ self-regulation techniques to modify their cognitive processes. It also refers to the managerial techniques they employ to regulate their educational journey. The three most important self-regulatory techniques are resource management, cognitive, and metacognitive techniques, according to [4]. The learning, memory, and comprehension processes employed by language learners are referred to as cognitive strategies. Put differently, language learners engage in processes that get new material ready for long-term memory storage and linkage and combination with existing knowledge. According to [5] metacognitive methods oversee, direct, and regulate cognitive strategies.

Language learners assess their comprehension and calculate the amount of time needed to study and overcome obstacles in order to achieve the desired result by using metacognitive methods. Resource management techniques make up the third self-regulation method. It shows how much time they require in order to utilize the allocated time as efficiently as possible [6]. Language learners who are good with time may take charge of their lives. Self-regulatory people can adjust and adapt the environment to a suitable one since they are aware of how environmental circumstances affect their accuracy during learning. Furthermore, [7] postulated that there are three cyclical phases of self-regulation: the stages of foresight, performance, and self-reflection.

Using SRSs can increase the creativity of EFL learners. According to [8], creativity is the process of bringing innovative concepts to life. Thinking and creating are the first steps in creativity, while innovation is the act of generating or putting an idea into practice. Teachers are imaginative but not creative if they have ideas but don’t follow through on them [9]. Notably, every concept ought to be tested in an educational setting to see whether or not it is effective. Regardless of whether an individual has produced any work in the past, creativity is frequently described as the capability to generate creatively [10].

In the context of education, learner creativity is the capability of students to make or bring into life something novel and innovative, whether it a novel way of learning material or a creative solution to an issue they encounter in class [11]. The following idea on the significance of creativity in instruction was put up by [12]: creative students have a higher chance of achieving the intended learning outcomes. This is partly because students who lack creativity are forced to employ outdated teaching methods that are ineffective for learning new material [13].

Applying SRSs can also develop motivation of EFL learners. It is often recognized that motivation has a major part in the effectiveness of language acquisition [14]. Long-term learning success depends on motivation, especially for young learners. Higher success is ultimately the result of more motivated students being more engaged in the learning process [15, 16]. posits that motivation to learn is comprised of three key components: the effort expended in pursuing a goal, the attitude maintained in the process, and the desire to reach a learning objective.

Pupils that are highly motivated usually have a great desire to succeed and a good outlook on life, and they put forth a lot of effort to do so. Lowly motivated learners, on the other hand, lack motivation to study, have unfavorable views toward the topic, and/or put up minimal effort to achieve their objectives [15]. All of these components—effort, desire, purpose, and attitude—are necessary to maintain motivation, and the loss of any one of them would suggest a lack of motivation. For instance, if a student wants to use language fluently but doesn’t put in the necessary effort, this objective won’t be accomplished and the student can be viewed as lacking motivation [17].

Pupils acquire English for both integrating and instrumental reasons. When students study for practical purposes, it’s because they see the language as valuable for achieving some sort of external goal [18]. This value might include studying for a future career, a college degree, or just passing an exam for EFL learners. However, studying for integrative goals necessitates a positive perception of the target culture as well as a desire to integrate [19]. The reason language learners who are studying for this aim are willing to acquire a language is that they wish to assimilate into the target group and they identify with that culture [20]. point out that pupils can approach learning in both instrumental and integrative ways, despite the fact that both techniques are frequently presented as mutually contradictory. In addition to their desire to become part of the target language use group, they may be motivated to study because of the potential advantages that come with learning the language.

In addition to motivation, self-efficacy of EFL learners can be affected by using SRSs. According to [21], “self-efficacy” refers to a person’s conviction in their ability to create real impacts and learn/apply manners at the appropriate levels. Instructional tactics that include goal-setting, progress feedback, modeling strategies, and self-evaluations of progress can boost self-efficacy and accomplishment. Additionally, by implementing these and other strategies in the classroom, instructors will support students’ sense of self-efficacy [22, 23]. state that learners with poor self-efficacy are those who think there is no connection between their actions and the results they experience, instead attributing their circumstances to other forces. They are less effective since they are not expected to put much effort into completing a task and are inclined to give up as soon as it is possible to do so. Because of this, Iranian EFL students who exhibit intrinsic motivation are more likely to be self-efficacious; in fact, high levels of intrinsic drive are positively correlated with self-efficacy.

Utilizing SRSs can also enhance WTC of EFL learners. The capacity to communicate in the target language has been seen as a central constituent of successful L2 acquisition in the field of language learning and has caused anxiety among L2 learners [24]. It has been assumed that obtaining communicative competence requires language learners to actively and meaningfully participate in classroom activities [25]. It has been suggested that a crucial factor in successful L2 communication is the inclination to engage and participate in classroom activities and conversations. This tendency, known as WTC, is defined as being prepared to engage in discourse with a certain person or people using an L2 at a given moment. According to [26], WTC is the yearning to begin a conversation when given the option in the setting of a L2.

According to [27], learners’ frequent use of second or foreign languages both within and outside of the classroom can be attributed to WTC. According to [28], WTC is the ultimate objective of education. According to [29], a learner’s willingness to take the lead in communicating with others in particular situations is a sign of their WTC. According to [30], fostering WTC in students—a crucial element of contemporary training in foreign or second languages—can have a profound impact on their communication abilities. Language teachers were encouraged to inspire their students to use the target language communicatively and authentically in a variety of conversational contexts by the introduction of the WTC idea in foreign language education [31].

Working on psychological factors that play a vital role in learning and teaching English language can be the significance of the study. While most previous studies examined the effects of SRS on EFL learners’ English language main skills such as writing and reading skills, this study tried to be different as it inspected the impacts of SRS on Chinese EFL learners’ motivation, self-efficacy, WTC, and creativity.

## Literature review

### Theoretical background

#### Self-regulation strategy

Based on [32], self-regulation is a psychological construct that is characterized as self-generated ideas, behaviors, and feelings that are planned and modified in response to performance feedback in order to accomplish self-established goals [33]. defined SRL in various contexts as goal-setting, preparation, strategy selection and application, self-evaluation, and self-monitoring. According to [34], SRL is an active and beneficial process in which pupils establish learning goals and then work to monitor, regulate, and control their motivation, cognition, and behavior, which are all influenced by and constrained by these objectives as well as the environmental context.

Furthermore, [35] described SRL as a dynamic learning process in which learners employ a variety of techniques to improve their behavior monitoring and cognitive abilities. Similar to this, [36] described SRL as a strategy to help students become autonomous so they may take charge of their own learning and problem-solving and become behaviorally, motivationally, and intellectually engaged. The practice of fostering student autonomy suggests that self-regulation is a skill that can be enhanced.

Self-regulation is one suggested strategy for encouraging pupils’ independence and autonomy. Academic self-regulation is seen by L2/EFL acquisition theorists as a more comprehensive concept than learning strategies, including reading strategies [37]. According to [38], self-regulation “describe[s] learners who learn for their own purposes in spite of often adverse circumstances.” Self-regulation is the capability to create and plan one’s own emotions, ideas, and actions, then modify them while performing in order to achieve one’s objectives. In a similar vein, SRL refers to learning that is based on students’ self-generated ideas and actions with an eye toward one’s learning goals. This includes self-regulation of motivation and emotion [39].

#### Self-efficacy

According to [40], self-efficacy is the belief in one’s own ability to plan and carry out the actions necessary to achieve certain performance goals. According to him, self-efficacy identifies people’s confidence in their ability to handle difficult jobs and use the necessary techniques to succeed in upcoming circumstances. Based on [41], self-efficacy also indicates how to control and manage learning objectives and persevere in completing tasks. It also establishes how resilient and nervous the student is about handling challenges [42]. identified four main strategies to increase self-efficacy: physiological and emotional states, vicarious experiences, social persuasion, and mastery experiences.

According to studies, positive psychology concepts like pride, enjoyment, optimism, resilience, grit, and engagement, well-being may all be aroused by self-efficacy. Since it increases students’ satisfaction, optimism, and pride in their academic achievements, [43] highlighted the growth of EFL learners’ self-efficacy in their academic successes [44]. contended that achievement, self-sufficiency, and self-efficacy are strongly correlated with EFL learners’ satisfaction. They maintained that knowing the target language, teachers’ encouraging methods, students’ self-assurance, and a favorable foreign language environment all had an impact on how much fun EFL learners have. According to [45], psychological well-being and self-efficacy are significantly correlated.

 [46] found that people who have greater levels of self-efficacy also tend to be more intrinsically motivated, set challenging goals for themselves, and maintain a strong commitment to their pursuits [47]. identified verbal persuasion, vicarious experiences, enactive mastery experiences, and an individual’s physiological and affective condition as the four main sources of self-efficacy beliefs. According to [48], the primary factor contributing to self-efficacy is active mastery experiences. Enactive mastery experiences, according to their explanation, are associated with an individual’s awareness of his or her own capacity to effectively do a particular activity based on prior successes. They stated that enactive mastery experiences are associated with people’s perceptions of their own abilities as well as the difficulty of the job and the amount of effort they would put in to complete it (51).

As the second source of self-efficacy, vicarious experiences, according to [49], are concerned with the social comparison of an individual’s performance to that of others with similar talents. According to [50], witnessing others’ similar talents can boost a person’s self-efficacy by confirming that their knowledge, skills, and methods are adequate. The third source of self-efficacy, verbal persuasion, is socially persuasive feedback—that is, remarks made by important people about one’s performance [47]. According to [51], positive remarks that highlight a person’s skills or accomplishments can boost self-efficacy. The fourth source of self-efficacy, an individual’s physiological and affective condition, is associated with their capacity to regulate their body’s and their emotions’ stress reactions (such as breathing, anxiety) in relation to their execution of tasks [52].

#### Creativity

According to [53], creativity is the ability to combine preexisting parts to generate novel and practical ideas. The main difference between creativity and innovation is therefore the generation of new ideas; nevertheless, innovation also necessitates the implementation of changes based on those ideas [54]. defined creativity as the capacity to respond to a given situation with meaningful solutions, innovative ideas, and well-thought-out methods.

It seems that there is a lot of interest in the study of creativity. Almost all prominent scientists explain this event in their own words. According to [55], the distinction between divergent and convergent thinking has probably had the most influence on our understanding of creativity’s evolution. While convergent thinking leads one to the correct, conventional option, divergent or creative thinking requires coming up with several unique answers and solutions [56]. defined creativity as the capacity to draw conclusions, develop hypotheses, evaluate and test ideas, convey discoveries, and be aware of problems, shortcomings, and gaps in knowledge.

Creativity is the state or capacity of creating, bringing into being, investing with a new form, generating by innovative aptitude, manufacturing, or bringing something new to life. Unquestionably, one of the most important ways that creativity manifests itself is via language [57]. Language usage, including comprehending and producing it, is a highly automated skill during early infancy. The majority of things that are said or heard are being spoken for the first time due to the very nature of language. Most of the things we say and hear are generated; they are not something we can recall by recollection. Language is stored in knowledge of speech sounds, word patterns, and building and word-stringing rules. Following the acquisition of these spontaneous skills and knowledge, language use gets almost completely creative and subconscious [58].

According to [59], acquiring a language requires the ability to both produce and understand new sentences that have never been heard or spoken before. This suggests that language includes a creative component. Every speaker of a language is able to create new sentences each time they speak and is also able to figure out new sentences that others have produced. We recognize that we should respect creativity more and make a conscious effort to include it into our daily training when we accept that everyone is creative and that creativity is an integral aspect of personality. Fortunately, like most other skills, creativity is one that can be improved. Furthermore, creativity provides educators with practical and inventive solutions that enable children to enjoy language acquisition and adapt language to cope with unexpected situations [60].

Creativity and language are closely connected human abilities. All creative undertakings, including artistic originality, scientific discovery, linguistic ingenuity, and more, are considered to follow a fundamental pattern [61]. However, many linguists agree that language is creative and that it possesses an intrinsic creative nature. Language competency enables speakers to combine words to form phrases, phrases to form sentences, and sentences to construct paragraphs, according to [62].

#### Learning motivation

Another study variable that is anticipated to be favorably impacted by SRSs is motivation [63]. defined motivation as the internal urges that initiate, lead, coordinate, amplify, terminate, and assess cognitive and motor activities in a constantly changing cumulative manner. These incentives enable the selection, operationalization, prioritization, and successful or unsuccessful execution of initial goals and aspirations. Various internal drives that motivate action toward objectives are included in other definitions of motivation [63]. As to [64] assertion, motivation serves as a construct that determines the success or failure of challenging undertakings.

To be motivated, according to [65], is to be inspired to carry out an action or engage in an activity. In contrast to uninspired students, who lack the desire and excitement to finish the assignments, motivated students are passionate and lively. Curiosity, interest, and a desire to do things are the key characteristics of motivated persons [66]. Furthermore, motivation requires maintenance; curiosity on its own is inadequate. More time and effort must be invested in order to reach a goal, and the required impact must be maintained [67]. defined motivation as the combination of a need or expectation, an action, a goal, and occasionally several kinds of reinforcement.

 [14] stated that motivated individuals put in a lot of effort to reach their goals, are tenacious in completing the tasks required to do so, have a strong desire to achieve their goals, enjoy the tasks required to do so, are persuaded to pursue their goals, and predict whether they will succeed or fail. When they are successful in some way, these people show high levels of self-efficacy and confidence in themselves. Their behaviors are supported by reasons, which serve as justifications and explanations [68].

 [69] enumerated a few factors that may impact students’ motivation. Among these essentials is achieving excellent marks; in fact, a student’s motivation may rise when they perform well in class. For students, achieving teacher objectives serves as an additional source of motivation. The professors’ feedback may also have an effect on the pupils’ motivation. Students will be more motivated if their professors go over their work and provide clear remarks about both their strong and weak points [69]. asserted that another source of motivation for pupils is material that is very relevant to their everyday lives. Considering the importance of strong motivation, we need to create an efficient learning environment that encourages students to learn languages.

#### WTC

Even though many language learners score highly on several language acquisition assessments, a large number of them seldom engage in L2 conversation. This problem demonstrates that there is an additional concept that stands between the ability to communicate and the ability to put this ability into practice [28, 70]. introduced the concept of WTC in the research on foreign language acquisition. Using an L2, they described this idea as being prepared to join the conversation at a certain moment with a specific person or people.

 [71] further said that the term “WTC” describes a learner’s cognitive preparation for using the target language in his communications. As the intention to communicate can lead to genuine communication behaviors, which increases foreign language competence, [72] viewed WTC as the primary goal of language acquisition. According to [24], WTC is a multifaceted construct that may explain, predict, and describe language learners’ communicative behavior in a L2. It includes emotional, social-psychological, linguistic, and communicative characteristics. The WTC of foreign language learners has been examined from trait-like, dynamic, and situational dimensions, in line with [70] model [73].

Self-confidence, motivation, and anxiety connected to learning a foreign language are all correlated with the trait-like or psychological dimension of WTC [74]. However, the contextual and social aspects of education, such as interlocutors, interactional themes, teachers, and cooperative peers, are referred to as the dynamic and situated dimensions of WTC. Many investigators have recently become interested in the topic of WTC [75].

Since the theory’s inception, researchers have started examining the ways in which individual factors like gender, age [76], anxiety related to learning a foreign language, and motivation for language acquisition can either directly or indirectly affect WTC. At the same time, researchers have started to see that WTC may be concentrated on dynamic factors like the function of online resources [77].

### Experimental studies

In this section, some empirical studies from some researchers were reported. The empirical studies were conducted on the effects of SRSs on English language skills and sub-skills of EFL learners. The In order to determine if teaching self-regulation improved the efficacy of a writing technique education program, [78] conducted a research. They contrasted teaching self-regulation and composition methods alone with teaching only composition strategies. The outcomes of their investigation demonstrated that pupils who had been taught self-regulation and composition techniques performed noticeably better, meaning that their writing assignments were of higher caliber.

The influences of teaching SRSs on Iranian EFL students’ metadiscoursal writing skills were examined by [79]. In order to do this, 50 EFL intermediate students studying English at an institution were chosen using a convenient random sample method. Students completed a pretest on metadiscoursal writing. Participants were instructed on self-regulated ways for intervention throughout the course of the next six sessions, and they were expected to adhere to the guidelines. The teacher evaluated each student’s development. They then completed a posttest, and a paired samples t-test was used to assess the data from the research equipment. The results showed that teaching the self-regulatory technique had a favorably meaningful effects on the metadiscoursal writing skills of Iranian EFL students, and it is recommended that teachers become more knowledgeable about the self-regulated strategy and its advantages.

The goal of [80] study was to find out how learning identity styles and SRL techniques affected the acquisition of English relative clauses (ERC). In this regard, 60 EFL learners were chosen, and one CG and one EG were given at random. Data were gathered using a pretest and a post-test. One-way analysis of variance and analysis of covariance were used to examine the data. The usage of SLS has a substantial impact on ERC, according to the ANCOVA data. All three identification types did not, however, appear to have a mediating effect in this study setting, according to the findings of the ANOVA test.

 [81] study looked at how SRL-based instructor feedback affected the writing performance and self-regulated techniques of EFL students. Seventy tertiary students from two concurrent intact English writing classes participated in the study, which was performed by the researchers. To gauge their progress in English writing abilities and their use of writing techniques for SRL, students completed a pre-test, an immediate post-test, and a delayed post-test using a questionnaire. The findings showed that both the writing performance and the reported usage of SRL writing methods by EFL student writers were positively impacted by the SRL-based feedback intervention. The treatment group also demonstrated improvement in goal-oriented monitoring, knowledge rehearsal, feedback handling, and interest development when it came to SRL writing strategies. Additionally, the intervention fostered the use of SRL strategies for text processing, motivational self-talk, idea planning, and emotional control.

 [82] explored how teaching self-regulation techniques affected Iranian EFL learners’ L2 reading comprehension and how it improved their ability to learn on their own. In this study, self-regulation techniques were taught to the EG (*N* = 35), while traditional training was the sole thing given to the CG (*N* = 35). For gauging the participants’ uniformity in terms of reading ability, they first finished the reading portion of the IELTS exam. Before and after the course, they also finished a reading assessment that was based on the course book. Before and after the race ended, the participants finished the LASSI as well. The findings showed that while students in both groups made considerable progress in their L2 reading comprehension, the EG’s growth was much greater. Regarding SRL, alone the EG’s pupils demonstrated a noteworthy improvement on the LASSI, demonstrating the beneficial effects of teaching self-regulatory techniques.

 [83] studied how Chinese students who weren’t majoring in English felt about their ability to write in English after using SRL methodologies. In order to do this, a quantitative approach was employed, which involved using two surveys to assess writing self-efficacy and the use of SRL methods before continuing to examine the link using correlational analysis. The findings indicate that students who do not major in English have a comparatively good attitude regarding using SRL writing skills and a reasonable level of confidence in their ability to write. Higher levels of writing self-efficacy are more likely to be attained by those who have favorable opinions toward the application of SRL writing tactics.

 [84] attempted to demonstrate the impact of SRSs on the autonomy of high school students studying vocabulary in the second grade. In order to do this, 40 of the 46 students from the two intact classes who were chosen via cluster sampling and classified as pre-intermediate ones took part in the study. Then, they were divided into two groups of 20 students each—the EG and the CG at random. For 10 sessions, members of the EG were required to use SRL techniques; in contrast, the CG continued with instruction as usual and received no special attention. The outcomes showed that there was a meaningful difference in learning autonomy between the individuals in the two groups.

The studies reviewed above indicate that using SRSs in EFL contexts and classes is beneficial for students and teachers. It has produced positive effects on EFL learners’ vocabulary learning, reading skill, and writing skill. In fact, majority of the studies were conducted on the effects of SRSs on English language main skills and sub-skills but few empirical studies were done domain in the domain of psychological variables involved in English language learning. Therefore, this study intended to cover this gap by posing the following four questions:

**RQ1.** Does integration of SRSs generate positive impacts on Chinese EFL learners’ language learning motivation?

**RQ2.** Does integration of SRSs generate positive impacts on Chinese EFL learners’ WTC?

**RQ3.** Does integration of SRSs generate positive impacts on Chinese EFL learners’ language self-efficacy?

**RQ4.** Does integration of SRSs generate positive impacts on Chinese EFL learners’ language creativity?

Four null hypotheses were formulated in the present research:

**RQ1.** The integration of SRSs does not generate positive impacts on Chinese EFL learners’ language learning motivation.

**RQ2.** The integration of SRSs does not generate positive impacts on Chinese EFL learners’ WTC.

**RQ3.** The integration of SRSs does not generate positive impacts on Chinese EFL learners’ language self-efficacy.

**RQ4.** The integration of SRSs does not generate positive impacts on Chinese EFL learners’ language creativity.

## Method

### Research design

This research utilized a quantitative between-group quasi-experimental design. Due to the absence of randomization this research used a quasi-experimental design. Two groups of EG and CG were included in this study. SRS was the independent variable while motivation, self-efficacy, WTC, and creativity were dependent variables in this study.

### Participants

The participants of this research were chosen from Chinese EFL students participating in EFL courses at two English language institutes in Guang Dong. Eighty male and female intermediate EFL learners between the ages of sixteen and twenty-seven were chosen as research participants using convenience sampling method. The chosen individuals were divided into two equal groups: the EG and the CG.

### Instruments

An OQPT (See Additional File 1) was the first instrument employed in this study to confirm that students’ competence level was intermediate. The OQPT is a popular and internationally recognized language proficiency exam that consists of 60 multiple-choice questions covering grammar, vocabulary, and reading comprehension. Individuals who score between 30 and 47 will be classified as intermediate according to the OQPT grading rubric. The reliability of this test was computed by using KR-21 (*r* =.82).

The study included a creativity questionnaire as its second tool. There were thirty-three-choice questions on this scale created by [85]. A three-point Likert scale was used to grade the questionnaire. Actually, the four components of creativity that this quiz assessed were fluidity, originality, adaptability, and expansion. The Cronbach’s alpha coefficient in the current investigation was 0.86.

The third tool applied in this investigation was a self-efficacy questionnaire [86, 86]. designed and validated a 30-item, 5-point Likert scale questionnaire to evaluate learners’ self-efficacy in a foreign language. Five constructs were identified from this questionnaire: (a) self-efficacy to finish an online course (items 1–8, alpha = 0.93); (b) self-efficacy to interact with classmates (items 9–13, alpha = 0.92); (c) self-efficacy to use tools in a course management system (CMS, items 14–19, alpha = 0.93); (d) self-efficacy to communicate with instructors in an online course (items 20–24, alpha = 0.94); (e) self-efficacy to interact with classmates for academic purposes (items 25–30, alpha = 0.93). The questionnaire’s overall Cronbach’s alpha reliability was recorded 0.88.

An English Learning Motivation Questionnaire, adapted from [87], was the second tool used in this investigation (ELMQ). It was a 21-item, six-point Likert scale questionnaire aims to identify important motivational elements relevant to the current study. These variables include instrumentality, integrativeness, attitudes toward the community and L2 speakers, and two criteria measures: the learners’ planned learning effort and preferred language. Due to their duplication and lack of relevance to the goals of the current investigation, some of the original questionnaire’s items were eliminated. According to [87], the original questionnaire’s reliability was assessed utilizing Cronbach’s alpha, which came out to be.78. Based on the data obtained, the reliability of the updated questionnaire employed in the current investigation was calculated using Cronbach’s alpha (*r* =.89).

The WTC Scale, created by [88], was the final metric used in this investigation. The purpose of the scale was to assess participants’ WTC in a variety of settings, including as meetings, group discussions, one-on-one interactions, and public speaking engagements. The twelve items on the scale included a variety of communication contexts and recipients, including friends, acquaintances, and strangers. The purpose of the scale was to gauge the participants’ inclination to communicate using the English language. For each of the 12 scenarios that were given, participants were asked to rate their willingness on a numerical scale from 0 (meaning “never”) to 10 (meaning “always”). The WTC scale, created by [88], was utilized in this research to assess respondents’ propensity to communicate in English with a variety of interlocutors and in a variety of communication circumstances. Strong internal consistency and reliability of the scale are indicated by the high Cronbach’s alpha coefficient attained in this study (*r* =.83). In this study, every scale listed above was utilized for both the pre- and post-test. The validity of all instruments was confirmed by two English experts in applied linguistics.

### Procedures and analysis

Based on the availability sampling approach, two groups of forty EFL learners were chosen at the start of the study. The dependent variables of the research—language learning motivation, WTC, self-efficacy, and creativity—were then pretested using questionnaires on the two groups. Then the course of treatment began. In actuality, fifteen sessions were needed to complete the treatment, which was one of the most crucial parts of the study process; the Book of Connect 4 was trained to both groups. While the EG received treatment by using SRSs to acquire language, the CG received instruction traditionally. The goal of the intervention was to examine how students’ knowledge of personal, behavioral, and environmental SRL techniques, as well as behavioral SRL strategies, affected their independence in vocabulary acquisition. In other words, the treatment was given to the EG students so they could learn how to apply SRSs. According to [89], an independent learner is someone who is willing to assume a significant amount of personal responsibility for their own education. They should plan their work, make decisions about their own learning, set realistic goals, assess and analyze their own work, and learn from their mistakes and successes in order to become better autonomous learners. They should also develop strategies for handling novel and unexpected situations. More significantly, self-directed learners are self-reflective about their own learning and voluntarily collaborate with peers to learn. Following the intervention, each group’s members received the four questionnaires to evaluate the impact of the training on their desire for language acquisition, WTC, self-efficacy, and creativity. Lastly, ANCOVA tests and independent sample t-tests were utilized to analyze the data.

## Findings

The Shapiro-Wilk test was utilized to see if the data were normally distributed. The findings displayed that the pre and post-tests scores for all dependent variables followed a normal distribution. Consequently, parametric statistics were utilized to analyze the data.

**Table 1:** Descriptive statistics of Pre-Tests for all defendant variables

	Groups	N	Means	Std. Deviations	Std. Error Means
WTC	CG	40	32.70	4.54	0.71
	EG	40	31.77	5.74	0.90
Motivation	CG	40	45.10	7.19	1.13
	EG	40	44.57	7.00	1.10
Creativity	CG	40	44.12	7.16	1.13
	EG	40	43.10	6.38	1.00
Self-efficacy	CG	40	63.15	13.99	2.21
	EG	40	65.07	14.78	2.33

The mean scores of both groups on all pre-tests are nearly identical, as Table 1 illustrates. These findings demonstrate that there was no statistically meaningful difference between the two groups’ responses on the WTC, self-efficacy, creativity, and motivation pre-tests. However, an independent samples t-test was performed to see if this difference is meaningful.

**Table 2:** Inferential statistics of Pre-Tests for all defendant variables

		F	Sig.	t	Df	Sig. (2-tailed)	Means Differences	Std. Error Differences			
WTC		2.02	0.15	0.79	78	0.42	0.92	1.15			
				0.79	74.07	0.42	0.92	1.15			
Motivation		0.17	0.67	0.33	78	0.74	0.52	1.58			
				0.33	77.94	0.74	0.52	1.58			
Creativity		0.78	0.37	0.67		0.50	1.02	1.51			
				0.67	76.98	0.50	1.02	1.51			
Self-efficacy		1.00	0.32	− 0.59		0.55	-1.92	3.21			
				− 0.59	77.76	0.55	-1.92	3.21			

Based on Table 2, it can be inferred that there was no significant difference between the two groups on any of the four pretests, since all Sig values are over 0.05. In fact, the two groups performed identically on the WTC, self-efficacy, motivation, and creative pre-tests.

**Table 3:** Descriptive statistics of both groups on the self-efficacy posttests

Groups	Means	Std. Deviations	N
CG	69.27	21.29	40
EG	77.90	23.95	40
Total	73.58	22.93	80

The EG’s mean score is 77.90, while the CG’s is 69.27, as indicated in Table 3. It appears that on the self-efficacy posttest, the EG outperformed the CG in terms of scores. The One-way ANCOVA test was employed in the following table to determine whether there was a substantial difference between the self-efficacy posttest of both groups:

**Table 4:** Inferential statistics of both groups on the self-efficacy posttests

Source	Type III Sum of Squares	Df	Mean Square	F	Sig.
Corrected Model	13562.34	2	6781.17	18.65	0.00
Intercept	1236.34	1	1236.34	3.40	0.03
Pretest	12074.53	1	12074.53	33.21	0.00
Groups	2107.63	1	2107.63	5.79	0.01
Error	27991.04	77	363.52		
Total	474763.00	80			
Corrected Total	41553.38	79			

Table 4 indicates that there was a substantial difference between the two groups’ self-efficacy posttest outcomes, with Sig being.00, less than 0.05. In actuality, on the self-efficacy posttest, the EG fared better than the CG. We can conclude that the students who had utilized SRSs performed better than the students who had not applied SRSs.

**Table 5:** Descriptive statistics of both groups on the creativity posttests

Groups	Means	Std. Deviations	N
CG	55.00	8.50	40
EG	71.70	12.91	40
Total	58.35	17.27	80

The descriptive data of the two groups on the creativity post-test are shown in Table 5. The EG and CG have respective means of 71.70 and 55.00. On the creative post-test, it appeared that the EG did better than the CG. The One-way ANCOVA test in the following table can be utilized to accept or reject this claim:

**Table 6:** Inferential statistics of both groups on the creativity posttests

Source	Type III Sum of Squares	df	Mean Square	F	Sig.
Corrected Model	14734.26	2	7367.13	64.11	0.00
Intercept	3322.05	1	3322.05	28.91	0.00
Pretest	476.46	1	476.46	4.14	0.04
Groups	13780.96	1	13780.96	119.93	0.00
Error	8847.93	77	114.90		
Total	295960.00	80			
Corrected Total	23582.20	79			

Table 6 depicts that Sig (0.00) is less than 0.05, indicating that there is a significant difference (*p* <.05) between the two groups. Because of the SRS training, the EG did better than the CG on the creativity posttest.

**Table 7:** Descriptive statistics of both groups on the WTC posttests

Groups	Mean	Std. Deviation	N
CG	34.45	6.05	40
EG	40.72	7.57	40
Total	37.58	7.50	80

Table 7 shows that the mean score for the EG is 40.72, whereas the mean score for the CG is 34.45. In the WTC posttest, it seems that the EG outwitted the CG. The One-way ANCOVA test was employed in the following table to see whether there was a meaningful difference between the WTC posttests of both groups:

**Table 8:** Inferential statistics of both groups on the WTC posttests

Source	Type III Sum of Squares	df	Mean Square	F	Sig.
Corrected Model	1008.67	2	504.33	11.26	0.00
Intercept	1445.83	1	1445.83	32.30	0.00
Pre	221.16	1	221.16	4.94	0.02
Groups	708.10	1	708.10	15.81	0.00
Error	3446.71	77	44.76		
Total	117481.00	80			
Corrected Total	4455.38	79			

Table 8 demonstrates that there were meaningful differences between the two groups’ WTC posttest results, with Sig being.00, less than 0.05. On the WTC posttest, the EG conducted better than the CG, as seen in the table.

**Table 9:** Descriptive statistics of both groups on the motivation posttests

Groups	Means	Std. Deviations	N
CG	55.72	7.83	40
EG	74.50	15.70	40
Total	60.11	19.01	80

Table 9 shows that the EG’s mean score is 74.50 while the CG’s mean score is 55.72. On the motivation posttest, it appears that the EG outperformed the CG in terms of scores. A one-way ANCOVA test was conducted in the following table (Table 10) to ensure that there was a significant difference between the motivation posttests of both EG and CG:

**Table 10:** Inferential statistics of both groups on the motivation posttests

Source	Type III Sum of Squares	df	Mean Square	F	Sig.
Corrected Model	18082.42	2	9041.21	66.34	0.00
Intercept	1986.04	1	1986.04	14.57	0.00
Pretests	1522.40	1	1522.40	11.17	0.00
Groups	16163.76	1	16163.76	118.60	0.00
Error	10493.56	77	136.28		
Total	317657.00	80			
Corrected Total	28575.988	79			

The aforementioned table (Table 10) illustrates that the difference in the motivation posttests of both EG and CG was statistically significant, with Sig being less than 0.05 at.00. It is true that, in the motivation posttest, the EG outwitted the CG.

Briefly speaking, the results indicate that the EG outdid the CG in the four post-tests of the study. The Sig values of the four post-tests are less than 0.00; therefore, there are significant differences between the EG and CG post-tests. SRS affected the motivation, self-efficacy, WTC, and creativity of Chinese EFL learners positively.

## Discussion and conclusion

This study looked at how SRSs affected the motivation, self-efficacy, WTC, and creativity of EFL learners as they learned English language. The study’s findings showed that SRS enhanced Chinese EFL learners’ WTC, creativity, self-efficacy, and desire for language acquisition. According to [78] study, the self-regulation strategy helped EFL learners write better, which is along with the findings of the current study. Furthermore, [79] attested to the beneficial effects of teaching SRSs on the metadiscoursal writing abilities of Iranian EFL learners.

Besides, [80] work, which demonstrated the beneficial effects of SRSs on the acquisition of ERC, validates our findings. Furthermore, the obtained outcomes are consistent with those of [81], who verified the positive impact of SRSs on EFL students’ writing abilities. Furthermore, this study’s findings concur with those of [82], who investigated how teaching SRSs affected the L2 reading skill of Iranian EFL students. The results demonstrated that although students in both groups improved significantly in their L2 reading comprehension, the EG’s growth was far more pronounced.

Additionally, the findings of this study are in agreement with those of [83], who investigated the use of SRSs in a Chinese setting and found that the use of SRSs increased students’ writing self-efficacy and that they also had a generally positive attitude toward employing SRL writing skills. Additionally, [84] provided evidence of the beneficial effects of SRSs on junior high school students’ autonomy. Furthermore, the results of this study are consistent with those of several other studies, including [90] and [91], which showed that vocabulary learning was significantly impacted by self-regulated techniques. As a result, when SRS training is used in the classroom, students can be given the chance to use the useful tactics that will benefit their academic performance.

The outcomes also broadly support the findings of [92], who came to the conclusion that self-regulation techniques might improve students’ involvement, attitudes toward creativity, and academic integration. Self-regulation techniques can help pupils become more resilient and adept at adjusting to social situations, according to [93]. High self-regulation among students leads to good learning. They have a strong desire to advance, especially academically. By putting SRSs into practice, students may control their objectives and course of action for academic success and maintain their motivation through challenging tasks [94]. The EG may have surpassed the CG due to the benefits listed for the SRSs.

There are also other justifications for the results obtained in our study. The teacher in the SRSs group switched from a standard method of instruction to a learner-centered one in which he gave more weight to the students’ preferences and pushed them to be more accountable for their own English language proficiency. It was urged of the EFL participants to actively shape their own education. Actually, learning became better, quicker, and more efficient as a result of the SRSs training. According to [95], the EG’s group’s tactics really helped EFL learners reach their full potential as learners and develop into flexible, autonomous learners.

Since SRSs include active engagement with the surrounding environment, they can produce priceless results in education and the learning process. Successful self-regulation consistently directs students’ goal-achieving tactics [96]. The goal of SRSs is to focus students’ attention on developing the necessary abilities for the tasks at hand, managing them, and producing worthy outcomes in those tasks, all of which have intrinsic value [97]. As a result, children who enhance their SRSs are able to regulate and control their thoughts, feelings, behaviors, and beliefs. As a result, improved outcomes in control and value judgments pertaining to academic activities may arise from this kind of training. Self-regulation training can help students learn how to manage their emotional motivation and behaviors during the learning process. Additionally, self-regulating students can assess and monitor their behaviors by self-judgment, self-observation, self-reaction, and self-control [98].

Numerous studies have shown how important it is for instructors to foster a welcoming classroom atmosphere in order to develop students’ self-regulation. For example, [99] claimed that in order to improve language learners’ academic self-regulation and vitality, teachers should foster an interactive and participatory environment, assign interesting assignments, stress the value of homework, and address other concerns pertaining to the learning environment of language learners [45]. discovered that language learners who exhibit strong self-regulation outperform those who exhibit low self-regulation in terms of their academic achievement, which is in line with the results of the current study. Put differently, good emotions are predicted by intellectual self-regulation [100]. also thought that students are motivated to participate in developmental activities, adverse environments, and future difficulties when they possess self-confidence and self-regulation. Adaptive functioning is linked to overcoming obstacles, such as having faith in one’s own ability to handle challenging external stimuli.

Learners of language who employ SRL techniques possess positive motivational views. It may be claimed that meaningful learning is the key to understanding this problem since it enables learners to integrate new information with previously learned frameworks and arrange their knowledge coherently. By creating connections between the acquired elements, this method facilitates the integration of new and fundamental knowledge and aids in the retention of information in language learners’ long-term memory [101].

Finally, it can be said that self-regulatory tasks and exercises in EFL textbooks can help students become more autonomous and motivated while also encouraging them to actively participate in the learning process and perceive themselves as agents of their own education. While doing this, it is also recommended that pre-service or in-service programs be established in order to systematically train EFL English teachers in SRSs. It is recommended that teachers familiarize themselves with the self-regulated method and its benefits in light of the findings of the literature and the current study [44].

Students’ WTC, inventiveness, self-efficacy, and enthusiasm to learn a language were all significantly increased by self-regulation training. These factors are critical for academic language accomplishment. The study’s outcomes suggest that SRSs may be used as an instructional strategy to boost students’ creativity, self-efficacy, WTC, and motivation. Teachers may create more engaging, thrilling, and intellectually challenging activities in the classroom by educating students about SRSs. Teachers may increase students’ motivation, WTC, self-efficacy, and creativity by including them in the learning process through their instructional strategies and classroom activities.

The results of this study could have an impact on educators, students, and material makers. Teachers’ and students’ perspectives on the significance of teaching and acquiring self-regulation abilities may shift if a clear grasp of the nature of the link between language acquisition and self-regulation is gained. Instructors may make an effort to provide students the domain, practical assistance, and strategic knowledge they need to be self-sufficient. Self-regulated skills are beneficial for students to acquire and may be integrated into their learning process to help them become more self-reliant and accountable for their own education.

The results of this study might have an impact on how instructional materials are created. In order to assist students learn English more successfully, material designers should concentrate on creating environments that will boost students’ willingness to read, write, and talk as well as reduce their fear and anxiety. The outcomes of this investigation have educational ramifications for both teaching and learning English. For language learners, being able to communicate is the ideal outcome. By better understanding language learners’ WTC, language teachers may improve their teaching strategies that accomplish this aim. The results also assist educators in expanding their understanding of the variables affecting language learners’ WTC. With this information, they may use SRSs to enhance language learners’ communicative behavior. Instructors may help students become more conscious of SRSs, teach them useful skills, and help them gain control over their thoughts, feelings, and actions. They can also help students broaden their perspective on the use of SRSs.

One of the study’s weaknesses is that age and gender were not taken into consideration. Further research is needed in order to generalize the findings to other age, gender, and proficiency groups. We suggest future researchers to include a large number of participants to boost the generalizability of their findings, as we were unable to include many in our study. We were unable to get qualitative data to enhance our findings; therefore, in order to strengthen the validity of their results, future researchers are encouraged to gather both quantitative and qualitative data. To be able to enhance findings by demonstrating “how” and “why” SRSs drive EFL learners to learn more effectively, future research should make use of mixed methodologies.

### Electronic supplementary material

Below is the link to the electronic supplementary material.

Supplementary Material 1

## Data Availability

The dataset of the present study is available upon request from the corresponding author.
